# Dysregulation of the vaginal microbiota in endometriosis and impact of endocrine disruptors: an exploratory case-control study

**DOI:** 10.3389/frph.2026.1978124

**Published:** 2026-10-23

**Authors:** Sophian Tricotteaux-Zarqaoui, Mariame Kabbour, Albane Vandecandelaere, Manon Soudain, Zeina Halbouty, Chafika Nadifi, Alessandra Cervino, Rosalie Cabry, Veronique Bach, Fatima Azzahra Lahlou, Moncef Benkhalifa, Hafida Khorsi-Cauet

**Affiliations:** 1 PERITOX-(UMR-I 01), UPJV/INERIS, UPJV, CURS, Amiens, France; 2 ART and Reproductive Biology Laboratory, University Hospital and School of Medicine, C.H.U. Sud, Amiens, France; 3 Laboratory of Chemistry-Physics and Biotechnology of Biomolecules and Materials (LCP2BM), Faculty of Science and Technology Mohemmadia, Hassan II University of Casablanca, Casablanca, Morocco; 4 Laboratory of Medical Analyses, Reproductive Biology, Labomac, Casablanca, Morocco; 5 Oncopathology Cancer Biology and Environment Laboratory, Mohammed VI Faculty of Medicine, Mohammed VI University of Health and Sciences (UM6SS), Casablanca, Morocco; 6 Health and Environment Laboratory, Ain Chock Faculty of Sciences, Hassan II University, Casablanca, Morocco; 7 PiLeJe Laboratories, Paris, France

**Keywords:** endocrine disruptor chemicals, endometriosis, reproductive health, vaginal dysbiosis, vaginal microbiota

## Abstract

**Background:**

Endometriosis is associated with chronic inflammation and immune dysregulation, yet its relationship with the vaginal microbiota remains poorly characterized. Emerging evidence suggests that endocrine-disrupting chemicals (EDCs) may further contribute to microbial imbalance. This study aimed to (1) characterize and compare vaginal microbiota composition between women with endometriosis and healthy controls, and (2) investigate associations between vaginal microbial profiles and self-reported EDC exposure.

**Methods:**

This exploratory case-control study was conducted between January 2024 and June 2025 at Amiens University Hospital. This analysis includes 25 surgically confirmed endometriosis cases (stage III-IV), and 25 controls, all of whom completed the study questionnaire. Vaginal samples were analysed by culture, Nugent scoring, and quantitative PCR targeting 11 bacterial species with total 16S rRNA as endogenous reference. Group comparisons were performed using the Mann–Whitney U test and Fisher's exact test. EDC exposure was assessed by structured questionnaire in a survey cohort (*n* = 261; endometriosis *n* = 137, controls *n* = 124) which included the 50 women of the microbiota analysis.

**Results:**

Nugent scoring showed more intermediate-flora profiles in endometriosis (45.5% vs. 30.0%) and comparable bacterial vaginosis profiles (13.6% vs. 15.0%; *p* > 0.05). Total bacterial 16S biomass was comparable between groups (*p* = 0.17), validating the normalization. Quantitative PCR identified significant depletion of *Atopobium vaginae* (*p* = 0.001) and *Lactobacillus crispatus* (*p* = 0.018), alongside a significant increase of *Lactobacillus gasseri* (*p* = 0.008) in endometriosis, while total *Lactobacillus* genus abundance remained unchanged (*p* = 0.26), suggesting a qualitative rather than quantitative shift. In the survey cohort, insecticide diffuser use was more frequent among endometriosis patients (29% vs. 16%; *p* = 0.02).

**Conclusions:**

This exploratory study identifies a qualitative restructuring of the vaginal *Lactobacillus* community in endometriosis, characterized by depletion of protective taxa and enrichment of a less protective species, distinct from classical bacterial vaginosis. By combining classical microbiology, molecular analysis and a self-reported exposure questionnaire in the same cohort, this study provides preliminary data on vaginal microbiota and potential EDC exposure in endometriosis, opening new perspectives for non-invasive diagnostic approaches and microbiota-targeted interventions.

## 1 Introduction

Endometriosis is an estrogen-dependent chronic inflammatory disease affecting 10%–12% of women at reproductive age worldwide. Infertility is a major consequence, with approximately 50% of infertile women having endometriosis. Diagnostic delay of 3 to 10 years remains common due to the lack of non-invasive tools, and the condition is disproportionately represented among women undergoing *in vitro* fertilization. While this pathology is characterized by ectopic growth of endometrial-like tissue outside the uterine cavity (1, 2), this condition is associated with systemic inflammation, adhesions, fibrosis, and symptoms like pelvic pain and subfertility (3–5). Despite its prevalence, long-standing recognition (6, 7), and negative impact on quality of life (8), its diagnosis remains challenging due to symptom heterogeneity. Recent guidelines advocate for non-invasive methods like imaging and biomarkers (9, 10), prompting investigations into alternative diagnostic approaches.

The pathogenesis of endometriosis is multifactorial, with theories including retrograde menstruation, coelomic metaplasia, and stem cell involvement (6, 11). Emerging research highlights the role of the vaginal microbiota (12), a low-diversity ecosystem dominated by *Lactobacillus* species (e.g., *L. crispatus*, *L. iners*, *L. jensenii*, *L. gasseri*), which maintain a protective acidic environment (pH 3.0–4.5) through lactic acid, H₂O₂, and bacteriocin production (13, 14). Dysbiosis, marked by reduced *Lactobacillus* dominance and increased pathogenic species, may contribute to chronic inflammation and oxidative stress which produce reactive oxygen species (ROS) that can potentially induce genome and epigenome disorders in endometriosis (15–17). Vaginal microbiota dysbiosis is also being investigated in gynecological oncology, notably in endometrial cancer, where higher vaginal alpha-diversity, enrichment of anaerobic species and depletion of Lactobacillus have been reported (18, 19). Yet, despite its central role in reproductive tract immunity and recent exploration in other gynecological diseases, the vaginal microbiota remains poorly characterized in endometriosis, representing a critical gap in current knowledge.

In reproductive pathologies, it is well documented that endocrine-disrupting chemicals (EDCs), such as pesticides and industrial pollutant compounds, interfere with fertility (20, 21), immune pathways and induce gut microbiota dysregulation (22, 23). Considering the similarities between gut and vaginal microbial ecosystems, it is hypothesized that EDCs could affect the vaginal microbiota. Chen et al. have reported that exposure to EDC reduces protective *Lactobacillus* strains and promote inflammation (24). EDCs can interfere with hormonal and immune pathways, potentially exacerbating endometriosis risk (25, 26). However, their specific impact on vaginal microbial imbalances in endometriosis remains largely unexplored.

This study objectives are (i) characterizing the vaginal microbiota of women with surgically confirmed endometriosis compared with healthy controls using complementary microbiological approaches (culture, Nugent scoring, qPCR), (ii) determining whether endometriosis is associated with a distinct dysbiosis pattern, different from classical bacterial vaginosis and assessing lifestyle and to assess (iii) environmental exposures, including self-reported EDC use, to explore potential links between environmental factors and vaginal microbial restructuring

## 2 Methods

### 2.1 Study design and participants

Women aged 18–45 years undergoing *in vitro* fertilization-embryo transfer were recruited between January 2024 and June 2025. Vaginal microbiota analyses were performed on 25 women with surgically and histologically confirmed endometriosis and 25 age-matched healthy controls. The planned recruitment target was 80 women with endometriosis and 80 controls. This article reports an exploratory analysis of the first 25 controls and 25 cases. Additional participants are being analysed separately. All participants included in the microbiota analysis had completed the questionnaire. Cases had surgically and histologically confirmed endometriosis staged III–IV by the revised ASRM classification (27). Controls were age-matched healthy volunteers with no history of endometriosis, chronic pelvic pain, or recent gynaecological infection. It should be noted that controls were not disease-free women but rather women seeking fertility treatment, who may present with other reproductive conditions unrelated to endometriosis. A flowchart summarizing screening, exclusions, and final enrolment is provided in Figure 1.

**Figure 1:**
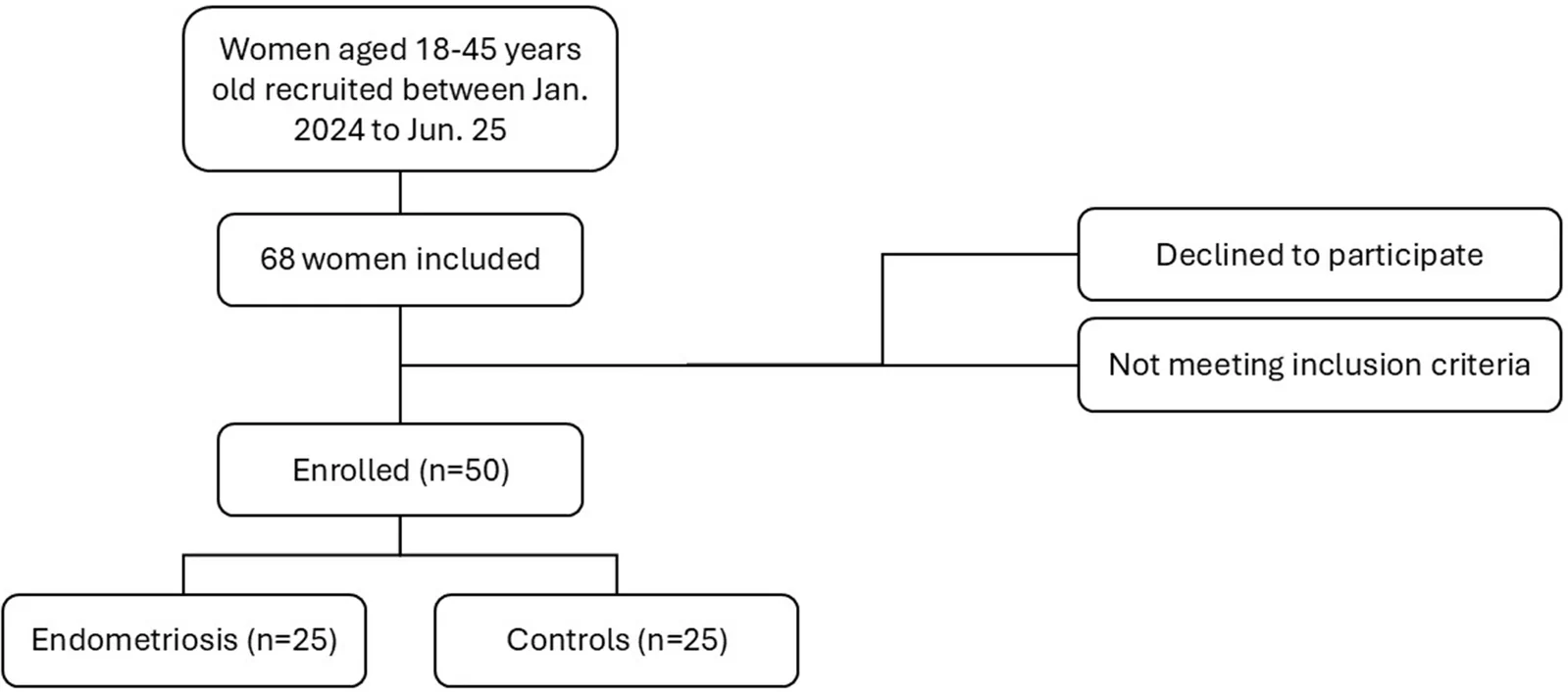
Participant flow diagram: endometriosis study recruitment, exclusions, and final analysed samples (endometriosis *n* = 25; controls *n* = 25).

### 2.2 Inclusion and exclusion criteria

Eligible participants were women aged 18–45 years with surgically and histologically confirmed endometriosis (stages III-IV) presenting with symptoms such as dysmenorrhoea or pelvic pain. Women were excluded if they were pregnant or breastfeeding, had received systemic antibiotics, vaginal antimicrobials, probiotics or for the medical management of endometriosis (combined oral contraceptives, progestins, or GnRH analogues) within the preceding three months. This exclusion criterion did not apply to IVF-related gonadotrophin stimulation: vaginal sampling was performed before the initiation of ovarian stimulation, and no participant was under exogenous gonadotrophin or progesterone support at the time of sampling.

### 2.3 Ethics and consent

The protocol was approved by the Institutional Ethics Committee of Amiens University Hospital (ID-RCB 2025-A02846-43). The questionnaire was reviewed and approved by the Institutional Ethics Committee of Université de Picardie Jules Verne (ID-RCB number: 2024-04-2) and was conducted in strict adherence to the principles outlined in the Declaration of Helsinki. Prior to enrolment, each participant was fully informed about the study's objectives and the role of the questionnaire. All participants provided written informed consent. Data handling complied with the Declaration of Helsinki and applicable data protection regulations.

### 2.4 Pre-sampling instructions and metadata

Participants were instructed to avoid sexual intercourse, vaginal douching, and intravaginal products for 72 h prior to sampling. Sampling was scheduled outside active menstruation. Menstrual phase (follicular, ovulatory, luteal) and contraceptive status were recorded. Additional metadata collected included age, BMI, parity, smoking status, recent antibiotic use, and recent sexual activity.

### 2.5 Sample collection and handling

Mid-vaginal swabs were collected using Sigma Transwab® flocked swabs (MWE, Corsham, Wilts, UK) in the following order: (1) swab for culture placed in Amies transport medium; (2) swab for molecular analysis placed in DNA stabilization buffer. Time from collection to laboratory processing, transport temperature, and storage conditions were recorded. Swabs for molecular analysis were stored at −20 °C until extraction (within 5 days).

### 2.6 Bacterial culture

Swabs for culture were processed within 4 h of collection. Samples were inoculated onto blood agar and chocolate agar (incubated at 37 °C in 5% CO₂ for 24–48 h), MacConkey agar (aerobic, 24–48 h), and Sabouraud agar (30 °C, up to 72 h). Anaerobic cultures were incubated in an anaerobic chamber or jar for up to 72 h. Growth was reported as presence/absence and semi-quantitatively (scant, moderate, heavy); where quantitative plating was performed, colony counts were expressed as CFU/mL. Reference strains and internal quality controls were included on each run.

### 2.7 Phenotypic and biochemical identification

Colonies were examined macroscopically and Gram-stained. Identification was performed using the Vitek 2 Compact system (bioMérieux, Marcy-l’Étoile, France) with GP/GN cards and ambiguous results were confirmed by API 20E (bioMérieux Marcy-l’Étoile, France) or MALDI-TOF (Bruker) when available. Species-level identifications were accepted at confidence scores ≥90%.

### 2.8 Microscopic evaluation using nugent scoring

Gram-stained smears were scored according to Nugent criteria (0–10). All slides were evaluated by a single experienced microbiologist blinded to clinical status. Discrepant or ambiguous smears were reviewed by the same reader a second time.

### 2.9 DNA extraction and storage

DNA was extracted using the Maxwell® RSC Buccal Swab DNA Purification Kit (Promega, Madison, WI, USA) with mechanical disruption using 200 µg of zircon beads in a FastPrep-24™ 5G (M.P. Biomedicals, LLC, Santa Ana, CA, USA), and the following modifications: bead-beating for 3 × 30 s at 6.0 m/s, proteinase K incubation at 56 °C for 20 min, and elution in 60 µL. Extraction blanks were included in each batch to monitor contamination. DNA concentration and purity were measured using a TECAN Infinite 200 Pro (Tecan Life Sciences, Switzerland).

### 2.10 Microbiological targets and rationale

Bacterial targets were selected *a priori* based on established ecological and clinical relevance within the vaginal microbiota. Four *Lactobacillus* species (*L. crispatus, L. iners, L. gasseri, L. jensenii*) were included because they define the major *Lactobacillus* dominated community state types and differ in their protective functions (28). *Gardnerella vaginalis* and *Atopobium (Fannyhessea) vaginae* were selected as hallmark taxa of bacterial vaginosis and Nugent intermediate flora. *Fusobacterium spp*. was included due to recent reports of endometrial infiltration and increased vaginal prevalence in endometriosis (29). Finally, *Enterococcus spp., Streptococcus spp.,* and *Escherichia coli* were selected as genera colonizing both the intestinal and genital tracts, to explore potential gut-vagina connections. Because the vaginal microbiota is dynamic and influenced by menstrual cycle phase, sexual activity, and hormonal status, sampling was scheduled outside menstruation, and standardized pre sampling instructions were provided.

### 2.11 qPCR and molecular assays

qPCR assays targeted total bacteria and species-/genus-specific 16S regions for key vaginal strains (primer sequences and assay details are available in Table 1). Reactions were run in technical triplicate with no-template controls and extraction blanks on each plate. Standard curves from serial dilutions of genomic standards were used to determine assay efficiency, linear range, and limits of detection. Relative abundances were calculated using the *ΔΔ*Ct method with total bacterial 16S (All Eubacteria) as the reference and the mean of the control group as the calibrator. Results are reported as fold-change 2^−*ΔΔ*Ct^ relative to controls.

**Table 1:** Primer sequences and amplicon details for qPCR assays used in this study. Columns Nucleotide Sequences: Forward primer (5′→3′); Reverse primer (5′→3′).

Bacteria phylum/species	Primer	Nucleotide Sequences (5′-3′)	Source
All Eubacteria	UniF	**F:** GTGSTGCAYGGYYGTCGTCA	(39) doi: 10.1017/S0007114508019880
	UniR	**R:** ACGTCRTCCMCNCCTTCCTC	
*Lactobacillus spp.*	16SrRNA-F	**F:** AGCAGTAGGGAATCTTCCA	(40) doi: 10.1128/AEM.01895-09
	16SrRNA-R	**R:** CACCGCTACACATGGAG	
*L. crispatus*	LcrisF	**F:** AGCGAGCGGAACTAACAGATTTAC	(33) doi: 10.1186/1471-2180-7-115
	LcrisR	**R:** AGCTGATCATGCGATCTGCTT	
*L. iners*	LInersF	**F:** GTCTGCCTTGAAGATCGG	
	LInersR	**R:** ACAGTTGATAGGCATCATC	
*L. jensenii*	LABJENTR2F	**F:** CCTTAAGTCTGGGATACCATT	
	LABJENRT2R	**R:** ACGCCGCCTTTTAAACTTCTT	
*L. gasseri*	LgassF	**F:** TGGAAACAGRTGCTAATACCG	
	LgassR	**R:** CAGTTACTACCTCTATCTTTCTTCACTAC	
*A. vaginae*	ATOVAGRT3F	**F:** GGTGAAGCAGTGGAAACACT	
	ATOVAGRT3R	**R:** ATTCGCTTCTGCTCGCGCA	
*G. vaginalis*	F-GV1	**F:** TTACTGGTGTATCACTGTAAGG	
	R-GV3	**R:** CCGTCACAGGCTGAACAGT	
*Enterococcus spp.*	EnterocF	**F:** AGAGAGTAAGGTCCGATTGAAC	(41) doi: 10.1016/j.ajog.2018.02.012
	EnterocR	**R:** GGTTGTTTCCCGTATTATGC	
*Streptococcus spp.*	StrepF	**F:** GTACAGTTGCTTCAGGACGTATC	(42) doi: 10.12659/msm.902237
	StrepR	**R:** ACGTTCGATTTCATCACGTTG	
*Fusobacterium spp.*	16SrRNA-F	**F:** CCCTTCAGTGCCGCAGT	(43) doi: 10.3892/etm.2019.8237
	16SrRNA-R	**R:** GTCGCAGGATGTCAAGAC	
*Escherichia coli*	ybbW-F	**F:** TGATTGGCAAAATCTGGCCG	(44) doi: 10.1016/j.watres.2017.08.032
	ybbW-R	**R:** GAAATCGCCCAAATCGCCAT	

### 2.12 Questionnaire and EDC exposure assessment

A structured questionnaire (Supplementary File 1) developed for this study, drawing on questions used in prior epidemiological surveys of environmental and occupational chemical exposure, captured demographics, reproductive history, household and occupational chemical exposure, dietary habits, and lifestyle factors. The questionnaire was presented to 302 participants; 261 were retained for analysis (endometriosis *n* = 137; controls *n* = 124), after excluding 41 incomplete submissions. All 50 women included in the vaginal microbiota analysis completed the questionnaire, and their responses were linked to their clinical records. The questionnaire data (*n* = 261) and the microbiota data (*n* = 50) are presented as two complementary analyses. Questionnaire data were anonymized prior to analysis. EDC-related variables collected through the questionnaire were self-reported and descriptive only, and no analytical verification or quantitative exposure assessment was performed.

### 2.13 Blinding and quality control

Laboratory personnel performing culture, Nugent scoring, DNA extraction, and qPCR were blinded to clinical groups. Extraction blanks and no-template qPCR controls were processed in parallel. Any strain detected in negative controls were reported and considered when interpreting low-abundance results.

### 2.14 Statistical analysis

Analyses were performed in R (v4.5.2; R Core Team, 2025). Continuous variables were assessed for normality using the Shapiro–Wilk test. Given the small sample sizes and non-normal distributions characteristic of qPCR relative quantification data, group comparisons were performed using the Mann–Whitney U test (two-sided; *α* = 0.05) throughout. Results are expressed as median relative quantification (RQ) with interquartile range [Q1–Q3]. Fold-change was calculated as the ratio of median RQ in the endometriosis group to median RQ in the control group.

For each qPCR target, samples were excluded based on pre-defined quality criteria applied independently to the target and reference amplicons: (1) fewer than two valid replicates (Ct < 45); (2) mean Ct of valid replicates > 40 cycles; or (3) coefficient of variation of valid replicates > 5%. A second pass of outlier exclusion applied a robust Z-score filter [Z = 0.6745 × (RQ−Median)/MAD; threshold |Z| > 3.5] calculated separately per group and per target, to identify samples with extreme RQ values attributable to partial DNA degradation or pipetting errors (30). These criteria were defined *a priori* and applied uniformly across all targets.

Categorical variables were compared using Fisher's exact test (two-sided). For questionnaire data, *χ*^2^ tests were used where expected cell counts were ≥5; Fisher's exact test was applied otherwise. Effect sizes are reported as fold-change for continuous qPCR data and as odds ratios with 95% confidence intervals for categorical comparisons. Given the exploratory nature of the study and the small sample sizes, *p*-values were not adjusted for multiple comparisons; all reported *p*-values are uncorrected and should be interpreted in this context. Effect sizes, 95% confidence intervals, and exact p-values are reported. A two-sided *p* < 0.05 was considered statistically significant.

## 3 Results

### 3.1 Vaginal microbiota analysis

#### 3.1.1 Participant characteristics

After screening and exclusions (Figure 2), the study analysed vaginal samples from 25 women with surgically and histologically confirmed endometriosis and 25 age-matched controls (total *n* = 50). The mean age was 33 years in the endometriosis group and 31 years in the control group. All participants were in the follicular phase (>day 7) at recruitment. Due to per-assay losses, culture and Nugent scoring were available for 42 samples (endometriosis *n* = 22; controls *n* = 20). DNA was successfully extracted from all 50 vaginal swabs (endometriosis *n* = 25; controls *n* = 25). Two extracts (one per group) did not meet spectrophotometric purity and yield acceptance criteria after re-extraction and were excluded from quantitative analyses; therefore, quantitative qPCR results are based on 48 extracts (endometriosis *n* = 24; controls *n* = 24). Due to variable amplification efficiency and DNA availability, the number of samples included per target ranged from 3 to 21 per group after per-target quality filtering. Demographic and questionnaire data used for EDC analyses derive from the larger survey cohort (*n* = 261) described below.

**Figure 2:**
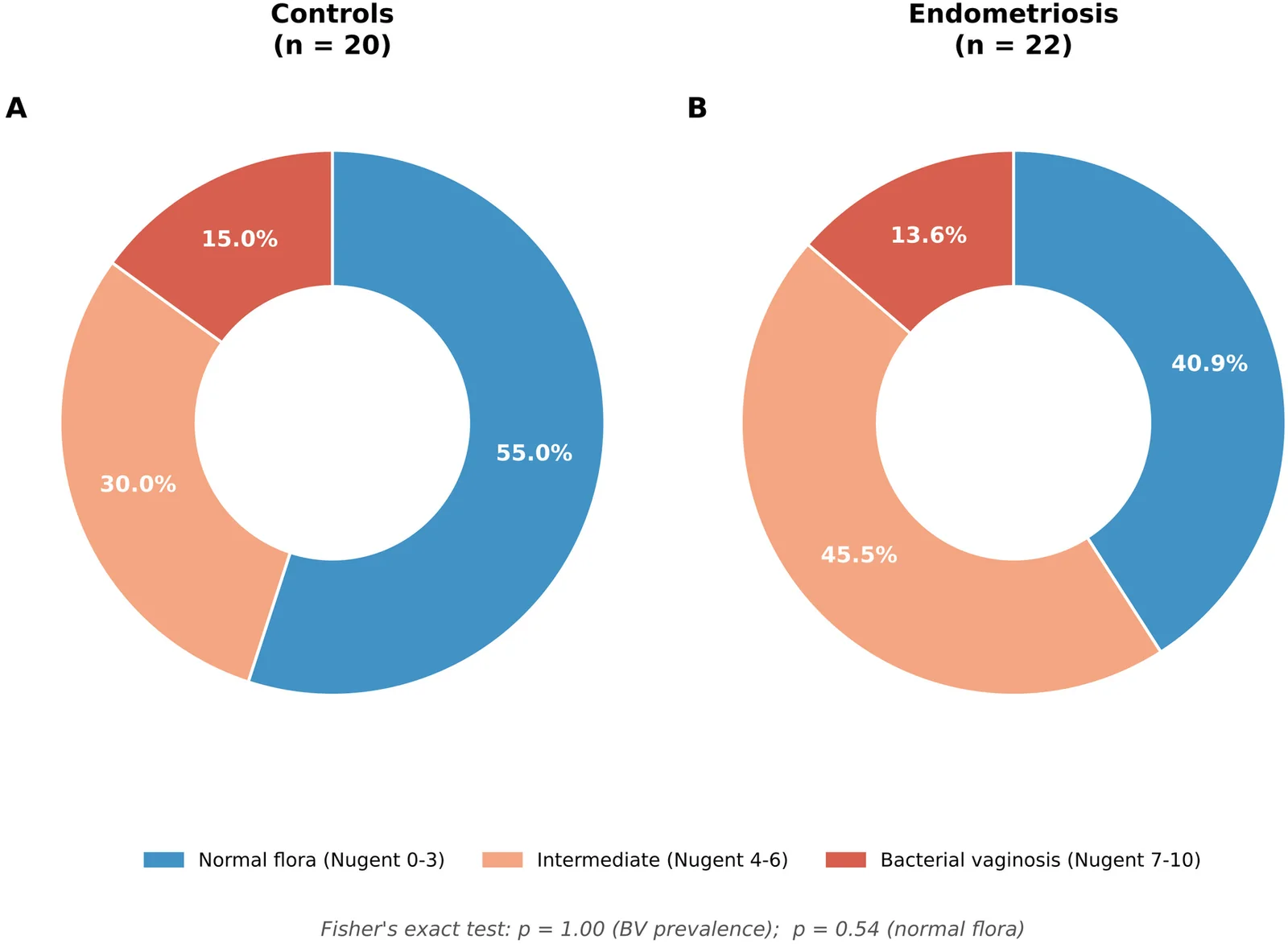
Distribution of vaginal microbiota profiles by nugent score in controls **(A)** and women with endometriosis **(B)** gram-stained smears were scored according to nugent criteria (0−10): normal flora (0−3), intermediate (4−6), bacterial vaginosis (7−10). Values are expressed as percentages. Fisher's exact test.

#### 3.1.2 Bacterial culture

Culture was attempted on vaginal swabs from all enrolled participants (endometriosis *n* = 25; controls *n* = 25). Viable bacteria were recovered from 42/50 swabs: 22/25 (88%) in the endometriosis group and 20/25 (80%) in controls. *Lactobacillus spp.* were isolated less frequently in the endometriosis group than in controls (22 vs. 20 positive cultures for any viable bacteria). Potentially pathogenic species including *Mobiluncus spp.*, *Enterococcus spp.*, and *Escherichia coli* were more commonly recovered from endometriosis samples. The difference in overall culture recovery between groups was not statistically significant (Fisher's exact test; *p* = 0.702).

#### 3.1.3 Nugent scoring

Gram-stained smears were available for 42 samples (endometriosis *n* = 22; controls *n* = 20) and were scored using the Nugent system (0–10). The distribution of Nugent categories by group is shown in Figure 2. In controls, 11/20 (55.0%) had normal flora (Nugent 0–3), 6/20 (30.0%) had intermediate flora (4–6), and 3/20 (15.0%) had scores consistent with bacterial vaginosis (7–10). In the endometriosis group, 9/22 (40.9%) had normal flora, 10/22 (45.5%) intermediate, and 3/22 (13.6%) bacterial vaginosis.

Comparisons by Fisher's exact test did not reach statistical significance in this cohort. The prevalence of Nugent scores consistent with bacterial vaginosis was comparable between groups (3/22 vs. 3/20; Fisher's exact two-sided *p* = 1.00). The prevalence of normal, *Lactobacillus*-dominant profiles did not differ significantly (9/22 vs. 11/20; Fisher's exact two-sided *p* = 0.54)

These data show a numerically higher proportion of intermediate-flora profiles in endometriosis (10/22 vs. 6/20; *p* = 0.35) and a lower proportion of *Lactobacillus*-dominant profiles (9/22 vs. 11/20; *p* = 0.54); however, the observed differences did not reach statistical significance in this cohort.

#### 3.1.4 DNA yield and QC

DNA was successfully extracted from all *n* = 50 vaginal swabs (endometriosis *n* = 25; controls *n* = 25). Spectrophotometric and fluorometric quality checks showed that two extracts (one endometriosis, one control) did not meet purity/yield acceptance criteria and were flagged for re-extraction. Re-extraction was attempted; when re-extraction failed to meet QC these two samples were excluded from quantitative qPCR analyses. Therefore, quantitative qPCR results reported below are based on 48 extracts (endometriosis *n* = 24; controls *n* = 24).

#### 3.1.5 qPCR performance

All qPCR assays met pre-defined plate quality thresholds (efficiency 90%–110%; R^2^ ≥ 0.99). No-template controls and extraction blanks were negative above the assay limit of detection in all accepted runs. Total bacterial 16S rRNA Ct values did not differ significantly between groups (median Ct: 16.7 in controls vs. 15.8 in endometriosis; Mann–Whitney U, *p* = 0.17; *n* = 17 controls/16 endometriosis), confirming comparable total bacterial biomass across groups and validating the use of relative abundance comparisons via the *ΔΔ*Ct method.

#### 3.1.6 Relative abundance by *ΔΔ*Ct (fold-change relative to controls)

Relative abundances were calculated using the 2^−*ΔΔ*Ct^ method with total bacterial 16S rRNA as the endogenous reference and the mean *Δ*Ct of included controls as the calibrator. Group comparisons were performed using the Mann–Whitney U test (two-sided; *α* = 0.05). Results are expressed as median RQ with interquartile range [Q1–Q3] and fold-change (FC = median RQ endometriosis/median RQ controls).

Species-level analysis of vaginal *Lactobacillus* community composition revealed a specific restructuring pattern in endometriosis, characterized by depletion of two key protective species and relative enrichment of a less protective species (Figure 3).

**Figure 3:**
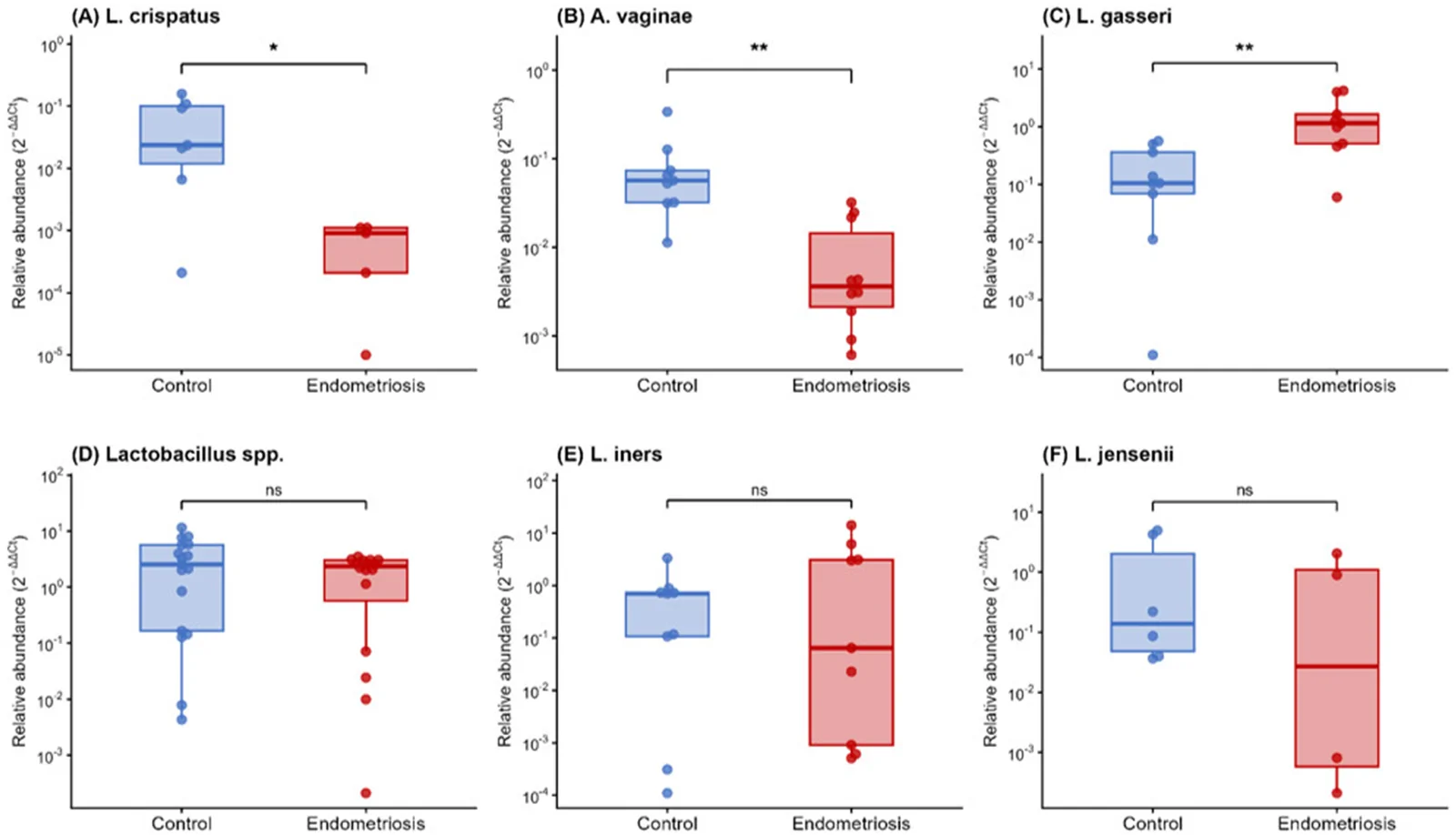
Vaginal *lactobacillus* community restructuring in endometriosis. Top row: **(A)** *L. crispatus* (*p* = 0.018, *); **(B)** *A. vaginae* (*p* = 0.001, **); **(C)** *L. gasseri* (*p* = 0.008, **). Bottom row: **(D)** *Lactobacillus spp.* total (*p* = 0.26, ns); **(E)** *L. iners* (*p* = 0.79, ns); **(F)** *L. jensenii* (*p* = 0.48, ns). Relative abundance by *ΔΔ*Ct method; total 16S rRNA reference. Box plots show median and IQR; individual values overlaid (beeswarm). Mann–Whitney U, two-sided. *Y*-axis: log10 scale.

#### 3.1.7 *Lactobacillus* community restructuring

*L. crispatus* was significantly reduced in endometriosis (Mann–Whitney U, *p* = 0.018; median RQ: 0.133 vs. 0.001; FC = 0.009; *n* = 7/5; Figure 3A). *L. crispatus* is the species most strongly associated with vaginal health, producing both D- and L-lactic acid and maintaining a low protective pH. *A. vaginae* was markedly reduced in endometriosis (*p* = 0.001; median RQ: 0.057 vs. 0.013; FC = 0.023; *n* = 9/10; Figure 3B), representing the strongest quantitative signal in the dataset, an approximately 44-fold lower median abundance. *L. gasseri* was significantly more abundant in endometriosis (*p* = 0.008; median RQ: 0.105 vs. 1.145; FC = 10.95; *n* = 9/9; Figure 3C). While a member of the *Lactobacillus genus*, *L. gasseri* is considered less protective than *L. crispatus*, producing predominantly L-lactic acid with weaker antimicrobial activity. Total *Lactobacillus genus* abundance did not differ significantly between groups (*p* = 0.26; FC = 0.96; *n* = 17/16; Figure 3D), indicating that the specific changes above reflect qualitative community restructuring rather than a simple loss of *Lactobacillus* biomass. *L. iners* (*p* = 0.79; FC = 1.13; *n* = 9/9; Figure 3E) and *L. jensenii* (*p* = 0.48; FC = 0.66; *n* = 6/4; Figure 3F) did not differ significantly between groups. Due to the high per-target exclusion rate for *G. vaginalis* after quality filtering (*n* = 6/3), no formal statistical comparison was possible.

#### 3.1.8 Gut-associated bacterial genera

Given published evidence of endometrial *Fusobacterium* infiltration in endometriosis, we investigated whether a vaginal signature could be identified in our cohort. We additionally examined three genera known to colonize both the gut and vaginal tract (*Staphylococcus*, *Enterococcus, E. coli*) as an exploratory gut-vaginal axis analysis.

*Fusobacterium spp*. showed a numerically lower median relative abundance in endometriosis (median RQ: 0.022 vs. 0.731 in controls; FC = 0.030; *p* = 0.21; *n* = 9/3; Figure 4A). *Staphylococcus spp.* showed no significant difference between groups (*p* = 0.14; FC = 0.209; *n* = 6/8; Figure 4B). *Enterococcus spp.* (*p* = 0.40; *n* = 4/3; Figure 4C) and *E. coli* (*p* = 0.86; *n* = 11/7; Figure 4D) were similarly non-significant. All results should be considered exploratory given the small per-target n, particularly for *Fusobacterium* (*n* = 3 in endometriosis) and *Enterococcus* (*n* = 3 in endometriosis).

**Figure 4:**
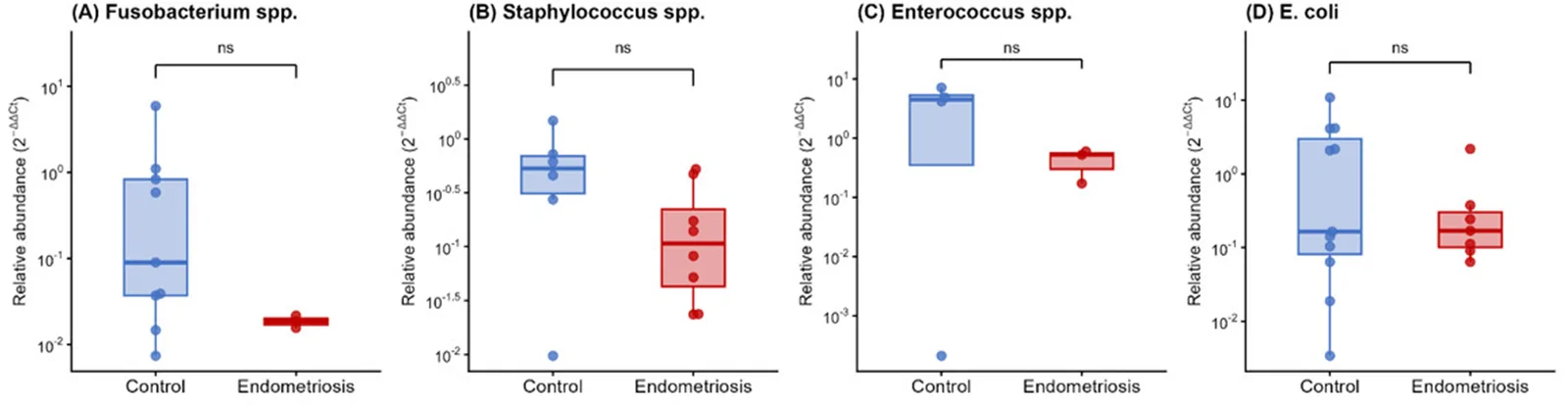
Gut-associated bacterial genera in the vaginal microbiota. **(A)** *Fusobacterium spp.* (*p* = 0.21); **(B)** *Staphylococcus spp.* (*p* = 0.14); **(C)** *Enterococcus spp.* (*p* = 0.40); **(D)** *E. coli* (*p* = 0.86). Exploratory analysis; all results non-significant. Relative abundance by *ΔΔ*Ct method; total 16S rRNA reference. Mann–Whitney U, two-sided. *Y*-axis: log10 scale.

*Streptococcus spp.* were significantly reduced in endometriosis (*p* = 0.025; FC = 0.13; *n* = 10/9). Given the exploratory nature of this study and the limited n. The biological significance of vaginal *Streptococcus* depletion in endometriosis is unclear and warrants investigation in larger cohorts.

### 3.2 Questionnaire and EDC associations

#### 3.2.1 Participant characteristics

Our study included 261 participants (137 with endometriosis, 124 controls). In Table 2, significant age distribution differences were observed (*p* < 0.001), with the control group skewed younger (35% aged 18–24 vs. 7% in the endometriosis group). Geographic distribution also differed significantly (*p* < 0.001), with higher representation from Nord (44%) and Pas-de-Calais (27%) in the endometriosis group. Notably, insecticide diffuser use was more common in the endometriosis group (29% vs. 16%, *p* = 0.02). Dietary adaptations were more frequent among endometriosis patients (34% vs. 9% of controls), with specific avoidance of gluten, lactose, or other allergens. Dairy consumption patterns also diverged (*p* < 0.05): 13% of the endometriosis group avoided dairy entirely (vs. 2% of controls), while controls consumed dairy more frequently.

**Table 2:** Demographic and behavioural characteristics of women with and without endometriosis in the French region of Hauts-de-France.

Criteria	Endometriosis (*n* = 137)	Controls (*n* = 124)	*p*-value
**Age Distribution**			**<0**.**001**
18–24	10 (7%)	44 (35%)	
25–30	35 (26%)	14 (11%)	
31–35	28 (20%)	9 (7%)	
36–40	33 (24%)	15 (12%)	
41–45	16 (12%)	10 (8%)	
46–50	9 (7%)	11 (9%)	
51–60	6 (4%)	15 (12%)	
>60	0	6 (5%)	
**Department Distribution**			**<0**.**001**
Aisne	9 (7%)	3 (2%)	
Oise	18 (13%)	9 (7%)	
Somme	13 (9%)	46 (37%)	
Nord	60 (44%)	9 (7%)	
Pas-de-Calais	37 (27%)	57 (46%)	
**Insecticide Diffuser Use**			**<0**.**02**
Yes	40 (29%)	20 (16%)	
**Adapted Diet**			**<0**.**05**
Any Adaptation	46 (34%)	11 (9%)	
Gluten-free	21 (15%)	3 (2%)	
Lactose-free	19 (14%)	2 (2%)	
Anti-inflammatory diet	13 (9%)	0	
Fodmap diet	3 (2%)	0	
Egg allergy	5 (4%)	1 (1%)	
Other diets	2 (1%)	1 (1%)	
**Dairy Product Consumption**			**<0**.**05**
Several times a day	11 (8%)	28 (23%)	
Daily	60 (44%)	66 (53%)	
Occasionally	48 (35%)	27 (22%)	
Never	18 (13%)	3 (2%)	
**Tobacco consumption**			**0**.**3**
Yes	26 (19%)	17 (14%)	
**Cigarette per day**			**0**.**6**
<3	5 (4%)	3 (2%)	
3–5	5 (4%)	1 (1%)	
6–10	6 (4%)	4 (3%)	
>10	10 (7%)	8 (6%)	
**Electronic cigarette**			**0**.**5**
Yes	11 (8%)	7 (6%)	
**Date of diagnosis**			
<6 months	10 (7%)	N/A	
<1 year	15 (11%)	N/A	
<2 years	13 (9%)	N/A	
<3 years	22 (16%)	N/A	
<5 years	22 (16%)	N/A	
>5 years	28 (20%)	N/A	
>10 years	27 (20%)	N/A	

## 4 Discussion

This exploratory study identified a specific pattern of vaginal microbiota alteration in women with endometriosis, characterized by a significant reduction of *A. vaginae* and *L. crispatus* and a significant increase of *L. gasseri*, consistent with a qualitative restructuring of the *Lactobacillus* community. A numerically higher prevalence of intermediate-flora profiles (Nugent 4–6) was additionally observed in the endometriosis group (45.5% vs. 30.0%; *p* = 0.35), with no increase in bacterial vaginosis-consistent profiles (13.6% vs. 15.0%; *p* = 1.00). An exploratory reduction of Streptococcus spp. (*p* = 0.025) was also detected, the biological significance of which remains unclear. These results are consistent with prior reports of microbial dysbiosis in endometriosis (31, 32) while also revealing patterns that diverge from classical bacterial vaginosis signatures.

### 4.1.1 *Lactobacillus* profiles and community stability

Total *Lactobacillus* genus abundance did not differ significantly between groups (Mann–Whitney U, *p* = 0.26; FC = 0.96), indicating that overall *Lactobacillus* population was broadly comparable. However, species-level analysis revealed divergent patterns within this genus. *L. crispatus* was significantly reduced in the endometriosis group (*p* = 0.018; FC = 0.009*). L. crispatus* is the species most strongly associated with vaginal health: it produces D- and L-lactic acid, maintains a low pH, and is inversely associated with BV and pelvic inflammatory disease (13, 28). Its specific depletion against a background of unchanged total *Lactobacillus* signal suggests a qualitative shift in community composition rather than a simple loss of *Lactobacillus* population.

In contrast, *L. gasseri* was significantly more abundant in the endometriosis group (*p* = 0.008; FC = 10.95). While a member of the *Lactobacillus* genus, *L. gasseri* is considered less protective than *L. crispatus*: it produces predominantly L-lactic acid, has weaker antimicrobial activity, and is associated with more transitional community states (33). An inverse relationship between *L. gasseri* and *L. crispatus* has been previously reported, and our data are consistent with a competitive replacement of the more protective species.

*L. iners* showed no significant difference (*p* = 0.79; FC = 1.13). *L. jensenii* did not differ significantly (*p* = 0.48; FC = 0.66), though a minor numerical trend toward lower abundance in endometriosis was observed.

### 4.1.2 *A. vaginae* depletion and divergence from classical BV

*A. vaginae* was markedly and significantly reduced in the endometriosis group (*p* = 0.001; FC = 0.023), representing the strongest quantitative signal in the molecular dataset. This finding is, at first sight, paradoxical: *A. vaginae* is typically considered a BV-associated organism, yet our cohort showed a higher prevalence of intermediate-flora profiles in endometriosis (45.5% vs. 30.0%, ns). This apparent contradiction may be reconciled by the polymicrobial nature of BV: the Nugent score does not distinguish *A. vaginae* from other BV-associated anaerobes such as *Mobiluncus spp.*, *Prevotella spp.*, or *Sneathia spp.* In endometrial cancer, higher vaginal alpha-diversity and enrichment of anaerobes such as *Prevotella, Anaerococcus* and *Mobiluncus* have been reported (19), although the comparison is limited by the marked differences in age and menopausal status between cohorts (mean age of 67 years in the endometrial cancer group). A such, intermediate Nugent profile with simultaneously reduced *A. vaginae* suggests that other anaerobic organisms may contribute to the dysbiotic signal, a pattern distinct from canonical BV and potentially specific to the endometriosis microenvironment.

### 4.1.3 Gut-associated and opportunistic genera

A landmark study by Muraoka et al. (29) demonstrated that *Fusobacterium* infiltrates the endometrium in 64% of women with endometriosis but fewer than 10% of controls, and identified a pathogenic mechanism whereby *Fusobacterium* is facilitating the establishment of endometriotic lesions. The same study also found higher *Fusobacterium* prevalence in vaginal swabs of affected patients, providing direct rationale for our vaginal qPCR analysis.

In our cohort, *Fusobacterium spp*. showed a numerically lower median abundance in endometriosis (FC = 0.030; *p* = 0.21), but this result is based on only a few valid endometriosis samples after quality filtering, critically insufficient to draw conclusions. The direction of the trend (higher in controls) is at odds with the Muraoka et al. findings, possibly reflecting true absence of vaginal enrichment, a type II error due to underpowering, or differential quality filtering between groups. A dedicated study with targeted *Fusobacterium* qPCR and larger n is required to clarify whether vaginal *Fusobacterium* enrichment accompanies the endometrial signature described by Muraoka et al.

Regarding the other gut-associated bacteria, *Streptococcus spp.* were significantly less abundant in the endometriosis group (*p* = 0.025; FC = 0.13). *Enterococcus spp.* did not differ significantly after objective quality filtering (*p* = 0.40). *Fusobacterium spp.* similarly showed no significant between-group difference (*p* = 0.21).

### 4.1.4 Possible biological interpretations

First of all, total bacterial biomass did not differ significantly between groups (*p* = 0.17), indicating that the four significant findings reported here reflect genuine shifts in vaginal microbial community composition rather than a non-specific reduction in overall bacterial load. This validates the use of the *ΔΔ*Ct normalization approach and strengthens the biological interpretation of the species-level differences observed.

Taken together, three consistent findings, which are depletion of *L. crispatus* and *A. vaginae*, and the increase of *L. gasseri*, describe a specific qualitative restructuring of the vaginal microbial community in endometriosis. An additional exploratory signal of *Streptococcus spp.* reduction (*p* = 0.025) was observed but cannot be biologically interpreted at this stage and warrants investigation in larger cohorts. Rather than a simple loss of all protective bacteria, the data suggest a qualitative shift: replacement of the most protective lactobacillus species (*L. crispatus*) by a less protective congener *(L. gasseri*), alongside depletion of *A. vaginae*.

Biologically, such changes could arise from altered local conditions in endometriosis inducing shifts in vaginal pH, mucosal secretions, oestrogen-mediated glycogen availability, immune mediators, or epithelial barrier function. The cross-sectional design prevents causal inference; microbiota alterations may be a cause, consequence, or epiphenomenon of disease.

A further mechanistic route linking environmental exposure to the genital ecosystem is the estrobolome, the collection of gut bacterial genes involved in oestrogen deconjugation. Bacterial *β*-glucuronidase activity can reactivate hepatically inactivated oestrogens in the intestinal lumen, enabling their reabsorption and modulating systemic oestrogen availability (34, 35). Because endometriosis is an oestrogen-dependent disease and vaginal epithelial glycogen (which conditions *Lactobacillus* colonisation) is oestrogen-sensitive, perturbation of the estrobolome could theoretically influence both lesion maintenance and vaginal community composition (14). EDCs can alter gut microbial communities and thus may indirectly affect oestrogen recycling via this axis, in addition to any direct receptor-mediated effects.

### 4.1.5 EDCs exposures and mechanistic hypotheses

Self-reported indicators of EDC exposure, notably insecticide diffuser use, were more common among participants with endometriosis in the survey cohort (29% vs. 16%, *p* = 0.02). These variables should be interpreted cautiously, as no analytical confirmation of exposure or association testing was performed. Although our questionnaire data are descriptive and circumstantial, this observation aligns with emerging reports linking occupational or environmental chemical exposures to elevated endometriosis risk (36–38). If confirmed by objective biomonitoring, EDCs could plausibly influence the vaginal ecosystem through endocrine modulation, immune dysregulation, or indirect effects mediated by the gut microbiota and mucosal barrier integrity (23). Mechanistic pathways worth testing include EDC-driven changes in local estrogen signalling, altered antimicrobial peptide expression, shifts in mucosal glycogen availability, and immune cell recruitment that together could reduce *L.crispatus* colonization or stability (14). These hypotheses remain speculative here because exposure data are self-reported and cross-sectional; prospective biomonitoring and experimental models are required to test causality.

The geographic distribution of survey respondents differed significantly between groups (*p* < 0.001), with higher response rates from endometriosis patients in Nord (44%) and Pas-de-Calais (27%). This distribution reflects differential questionnaire response rates across departments rather than a designed spatial sampling strategy and should be interpreted with caution. Nevertheless, this data is contextually noteworthy in the context of Hauts-de-France, a region known for its intense agricultural activity, with heavy pesticide use. Several studies have suggested a potential link between chronic exposure to pesticides and the risk of endometriosis. In France, large quantities of EDCs are used in agriculture, particularly in the form of pesticides. In 2017, 68,000 tonnes were purchased, placing France first in Europe and third worldwide. EDCs are recognised for their genotoxicity and neurotoxicity, notably affecting brain development (20). This study provides valuable insights into the relationship between endometriosis and exposure to endocrine disruptors. These findings highlight the need for further research to elucidate the underlying mechanisms and to develop effective prevention and treatment strategies.

### 4.1.6 Limitations and perspectives

Although exploratory in nature, this study provides an important first step toward characterizing vaginal microbiota alterations in endometriosis. The sample size (*n* = 50) was intentionally designed to enable detailed molecular and classical microbiological analyses; however, larger, adequately powered cohorts will be essential to validate these findings and detect more subtle microbial shifts. Our study population was drawn from the Hauts-de-France region, an area with intensive agricultural activity and elevated pesticide use. While this may limit generalizability, it also offered a unique opportunity to investigate microbiota patterns in a population with potentially higher environmental exposure, thereby strengthening the rationale for examining endocrine-disrupting chemicals in relation to reproductive health.

EDC exposure was assessed through structured self-reported questionnaires, which provided valuable insight into lifestyle and environmental factors but did not allow objective quantification of exposure. Future studies incorporating biomonitoring approaches (e.g., urinary or serum measurements) will be critical to refine exposure–microbiota associations. Likewise, our targeted qPCR strategy enabled precise quantification of key vaginal taxa, but broader community profiling using high-throughput sequencing will be necessary to capture the full complexity of microbial restructuring in endometriosis. Despite these considerations, our findings open several promising avenues for future research. Ongoing work in our laboratory includes high-throughput 16S rRNA gene sequencing of the endometrial microbiota to identify disease-specific microbial signatures, alongside an investigation of the gut-vaginal-endometrial microbiota axis and its role in endometriosis, as well as the evaluation of circulating cell-free DNA (cfDNA) as a potential non-invasive biomarker for endometriosis diagnosis and monitoring.

## 5 Conclusion

This exploratory study identifies a specific pattern of vaginal microbiota restructuring in women with endometriosis: depletion of the keystone protective species *L. crispatus* and *A. vaginae* and increase of the less protective *L. gasseri*. An exploratory reduction of *Streptococcus spp*. was additionally observed and warrants confirmation in larger studies. Importantly, the reduction of *A. vaginae* in a context of increased intermediate-flora profiles argues against a classical BV pattern and suggests a distinct form of microbial dysbiosis specific to endometriosis. While hypothesis-generating, these results provide a molecular foundation for larger studies incorporating full metagenomics, objective EDC biomonitoring, and comprehensive clinical phenotyping.

*Abbreviations are listed in alphabetical order. All abbreviations are defined at first use in the manuscript text.*

## 7 Additional requirements

### 7.1.1 Ethical approval and consent to participate

For additional requirements for specific article types and further information please refer to ‘Article types’ on every Frontiers journal page.

This study was carried out at the Amiens University Hospital, in Reproductive Medicine Department. ‘PESTIENDOMICRO’ protocol was approved by Institutional Ethical Committee on 28/02/2026 (ID-RCB number: 2025-A02846-43).

The questionnaire was reviewed and approved by the Institutional Ethics Committee of Amiens University Hospital (ID-RCB number: 2024-04-2) and was conducted in strict adherence to the principles outlined in the Declaration of Helsinki. Prior to enrolment, each participant was fully informed about the study's objectives and the role of the questionnaire.

Participation in this study was entirely voluntary, with individuals free to withdraw at any point without facing any consequences. Prior to enrolment, participants received a comprehensive explanation of the study's objectives, procedures, potential risks, and anticipated benefits. To ensure privacy, all data collected were anonymized, and no information was retained that could directly or indirectly identify any participant. The study protocol was designed to prevent any linkage between personal identities and the anonymized data, meaning no correspondence table or identifying code was used. As a result, the data are not classified as personal under applicable data protection standards

All procedures performed in studies involving human participants were in accordance with the ethical standards of the institutional and/or national research committee (ID-RCB number: 2025-A02846-43) and with the 1964 Helsinki Declaration and its later amendments or comparable ethical standards.

## Data Availability

The raw data supporting the conclusions of this article will be made available by the authors, without undue reservation.
