# Supplementary material for: Dysregulation of the vaginal microbiota in endometriosis and impact of endocrine disruptors: an exploratory case-control study
Source: Front Reprod Health. 2026 Oct 23;8:1978124. doi: 10.3389/frph.2026.1978124 (PMC13739678; doi:10.3389/frph.2026.1978124)
Supplement: Supplementary file 1 [file Supplementaryfile1.docx]

**ASSESSMENT OF ENDOCRINE DISRUPTOR EXPOSURE IN WOMEN WITH ENDOMETRIOSIS**

Endometriosis is a chronic systemic inflammatory disease affecting 10% of women worldwide. This condition impacts the quality of life of those affected every month through pain and/or infertility. To date, this common disease remains poorly understood.

This questionnaire aims to identify differences in exposure to endocrine-disrupting chemicals, particularly pesticides, between women with and without endometriosis. It will also allow us to observe potential changes in the genital microbiota (vaginal and uterine) that may be involved in endometriosis.

To this end, this questionnaire will begin with general questions, followed by more specific questions about your place of residence, diet, products used on a daily basis (household products, cosmetics), lifestyle habits, and some more personal questions about your gynaecological conditions.

Completing this questionnaire will take approximately 10 minutes. Responses are anonymous and comply with the criteria of the No-Interventional Ethics Committee.

Finally, to meet the study eligibility criteria, you must reside in the Hauts-de-France region (Aisne, Somme, Oise, Nord, Pas-de-Calais).

Your contribution is invaluable and will help us demonstrate the environmental impact on this condition. Thank you in advance for your participation!

| **GENERAL INFORMATION** | | |
| --- | --- | --- |
|  |  | |
| What is your age? |  | |
|  |  | |
| **What is your highest level of education?** |  | |
| ☐ No diploma / Junior high school certificate | ☐ General baccalaureate | |
| ☐ Vocational certificate (CAP, BEP) | ☐ Bachelor’s level (2–3 years post-baccalaureate) | |
| ☐ Technological or vocational baccalaureate | ☐ Master’s / PhD / Grandes Écoles | |
|  |  | |
| **Do you have any medical condition?** | ☐ Yes | ☐ No |
|  |  | |
| **If yes, which one/please specify:** |  | |
|  |  | |
|  | | |
|  |  | |
| **Do you take medication on a daily basis?** | ☐ Yes | ☐ No |
|  |  | |
| **If yes, which one/please specify:** |  | |
|  |  | |
|  | | |

| **YOUR CURRENT OCCUPATION** | | |
| --- | --- | --- |
|  |  | |
| What is your occupation? |  | |
|  |  | |
| Does your occupation expose you to chemical products? | ☐ Yes | ☐ No |
|  |  | |
| What is/was your mother’s occupation? |  | |
|  |  | |
| What is/was your father’s occupation? |  | |
|  |  | |
| If you live with someone, what is their occupation? | | |
|  | | |
|  | | |
|  |  | |
| **Is this your partner/spouse?** | ☐ Yes | ☐ No |

| **YOUR PLACE OF RESIDENCE** | | | | | | |  |
| --- | --- | --- | --- | --- | --- | --- | --- |
|  | |  | | | | |  |
| In which department do you currently live? | |  | | | | |  |
| ☐ Aisne | | ☐ Nord | | | | |  |
| ☐ Oise | | ☐ Pas-de-Calais | | | | |  |
| ☐ Somme | |  | | | | |  |
|  | |  | | | | |  |
| Since when have you been living in this department? | |  | | | | |  |
|  | |  | | | | |  |
| Where do you live? | | ☐ In a city/town | | | ☐ À la campagne | |  |
|  | |  | | | | |  |
|  | **YOUR PLACE OF RESIDENCE** | | | | | | |
|  |  | |  | | | | |
|  | Are there cultivated fields around your home? | | ☐ Yes | | | ☐ No | |
|  |  | |  | | | | |
|  | Are there industrial facilities near your home? | | ☐ Yes | | | ☐ No | |
|  |  | |  | | | | |
|  | Are there motorways, railways, or airports near your home? | | ☐ Yes | | | ☐ No | |
|  |  | |  | | | | |
|  | In which department did you grow up? | |  | | | | |
|  | ☐ Aisne | | ☐ Nord | | | | |
|  | ☐ Oise | | ☐ Pas-de-Calais | | | | |
|  | ☐ Somme | | ☐ Other |  | | | |
|  |  | |  |  | | | |
|  | Are there cultivated fields around your home? | | ☐ Yes | | | ☐ No | |
|  |  | |  | | | | |
|  | Are there industrial facilities near your home? | | ☐ Yes | | | ☐ No | |
|  |  | |  | | | | |
|  | Are there motorways, railways, or airports near your home? | | ☐ Yes | | | ☐ No | |

| **YOUR DIETARY HABITS** | | |
| --- | --- | --- |
|  |  | |
| Do you consume more than 50% of products from organic farming? | ☐ Yes | ☐ No |
|  |  | |
| Do you rinse fruits and vegetables before eating them? | ☐ Yes | ☐ No |
|  |  | |
| Do you peel non-organic vegetables? | ☐ Yes | ☐ No |
|  |  | |
| Do you frequently consume processed foods? (*Plats cuisinés, gâteaux industriels, pizzas surgelées, etc*) | ☐ Yes | ☐ No |
|  |  | |
| Do you follow a specific diet? (*Allergie, intolérance au gluten, régime végétarien...*) | ☐ Yes | ☐ No |
|  |  | |
| If yes, which one/please specify: |  | |
|  |  | |
| Do you drink tap water? |  | |
| ☐ Every day | ☐ Sometimes | |
| ☐ Often | ☐ Never | |
|  |  | |
| Do you use plastic containers to store food? | ☐ Yes | ☐ No |
| **YOUR DIETARY HABITS** | | |
|  |  | |
| Do you heat plastic containers in the microwave? | ☐ Yes | ☐ No |
|  |  | |
| What type of kitchen utensils do you mainly use? | | |
| ☐ Wood or metal | ☐ Plastic | |
|  |  | |
| What type of cookware do you use? |  | |
| ☐ Non-stick coating (Teflon) | ☐ Inox/ acier/ fonte/ aluminium/ céramique | |
|  |  | |
|  |  | |
| How often do you consume the following foods? | | |

|  | Plusieurs fois par jour | Tous les jours | De temps en temps | Never |
| --- | --- | --- | --- | --- |
| Soy or soy-based products | ☐ | ☐ | ☐ | ☐ |
| Seafood / fish | ☐ | ☐ | ☐ | ☐ |
| Fruits / Vegetables | ☐ | ☐ | ☐ | ☐ |
| Céréales ou produits a base de céréales  (*riz, semoule, pates, biscottes…*) | ☐ | ☐ | ☐ | ☐ |
| Dairy products | ☐ | ☐ | ☐ | ☐ |
| Œuf | ☐ | ☐ | ☐ | ☐ |
| Meat | ☐ | ☐ | ☐ | ☐ |

| **HOUSEHOLD MAINTENANCE** | | |
| --- | --- | --- |
|  |  | |
| How often do you ventilate your home? |  | |
| ☐ Once a day | ☐ < 3 fois par semaine | |
| ☐ > 3 fois par semaine | ☐ Never | |
|  |  | |
| Do you use air fresheners or room sprays? | ☐ Yes | ☐ No |
|  |  | |
| Where do you live? | ☐Oui | ☐ Non |
|  |  | |
| Do you use candles on a daily basis? | ☐Oui | ☐ Non |
|  |  | |
| Do you use insecticide products? (*Antiparasitaires, diffuseurs antimoustiques, …*) | ☐Oui | ☐ Non |
|  |  | |
| Do you use household products with an eco-label or homemade cleaning products? | ☐Oui | ☐ Non |
|  |  | |
| Do you wear gloves when cleaning? | ☐Oui | ☐ Non |
|  |  | |
| Do you wash new clothes before wearing them for the first time? | ☐Oui | ☐ Non |

| **COSMETICS** | |
| --- | --- |
|  |  |
| How often do you use nail polish or hair dye? | |
| ☐ Every day | ☐ Sometimes |
| ☐ Often | ☐ Never |
|  |  |
| How often do you wear make-up? |  |
| ☐ Every day | ☐ Lors d’occasions spécifiques uniquement |
| ☐ 5 days per week | ☐ Never |
| ☐ < 3 jours par semaine |  |
|  |  |
| Do you choose your cosmetics (*maquillage, gel douche, déodorant*) selon le label biologique ? | |
| ☐ Yes |  |
| ☐ Non |  |
|  |  |
| What type of menstrual protection do you use? | |
| ☐ Sanitary pad | ☐ Tampon |
| ☐ Menstrual cup | ☐ Period underwear |

| **SMOKING AND ALCOHOL** | | |
| --- | --- | --- |
|  |  | |
| Do you smoke? | ☐ Yes | ☐ No |
|  |  | |
| If yes, on average, how many cigarettes do you smoke per day? | | |
| ☐ < 3 cigarettes | ☐ Entre 6 et 10 cigarettes | |
| ☐ Between 3 and 5 cigarettes | ☐ > 10 cigarettes | |
|  |  | |
| Do you use an e-cigarette? | ☐ Yes | ☐ No |
|  |  | |
| If yes, do you consume nicotine? | ☐ Yes | ☐ No |
|  |  | |
| For how long have you been smoking? |  | |
|  |  | |
| If you have quit smoking, for how long did you smoke? |  | |
|  | | |
| Are you exposed to passive smoking from those around you? | ☐ Yes | ☐ No |
|  |  | |
| Do you consume alcohol? |  | |
| ☐ Every day | ☐ Once a week | |
| ☐ Once a month | ☐ Never | |
|  |  | |
| When you consume alcohol, how many drinks do you have? | | |
| ☐ 1 drink | ☐ > 5 verres | |
| ☐ > 2 verres |  | |
|  |  | |
| Do you consume cannabis? |  | |
| ☐ Every day | ☐ Once a week | |
| ☐ Once a month | ☐ Never | |

| **YOUR LEISURE ACTIVITIES** | | |
| --- | --- | --- |
|  |  | |
| Is gardening one of your leisure activities? | ☐ Yes | ☐ No |
|  |  | |
| Do you use pesticides to protect your garden? | ☐ Yes | ☐ No |
|  |  | |
| Do you have indoor plants? | ☐ Yes | ☐ No |
|  |  | |
| Do you use pesticides to protect your plants? (*Fertilisers*) | ☐ Yes | ☐ No |
|  |  |  |
| Is DIY/home improvement one of your leisure activities? (*Solvents, adhesives*) | ☐ Yes | ☐ No |
|  |  |  |
| Are you regularly in contact with animals treated with antiparasitic treatments (f*lea treatments, tick treatments, dewormers*) ? | | |
| ☐ Every day | ☐ Sometimes | |
| ☐ Often | ☐ Never | |

| **PREGNANCY AND FERTILITY** | | |
| --- | --- | --- |
|  |  | |
| Do you have children? | ☐ Yes | ☐ No |
|  |  | |
| Do you wish to have children? | ☐ Yes | ☐ No |
|  |  | |
| Do you have difficulties conceiving? | ☐ Yes | ☐ No |
|  |  | |
| Are you currently undergoing Assisted Reproductive Technology (ART)? | | |
| ☐ Yes | ☐ No | |
|  |  | |
| Was/were your pregnancy/pregnancies achieved through Assisted Reproductive Technology (ART)? | | |
| ☐ Yes | ☐ No | |
|  |  | |
| Did you undergo ART after having achieved natural pregnancies? | | |
| ☐ Yes | ☐ No | |
|  |  | |
| Do you have a gynaecological condition? (*Other than endometriosis*) | | |
| ☐ Yes | ☐ No | |
|  |  | |
| If yes, which one/please specify: |  | |
|  |  | |
|  | | |
|  |  | |
| Do you have endometriosis? | ☐ Yes | ☐ Yes |
|  |  | |
|  |  | |
| **If the answer is NO, the questionnaire is now over.**  **Thank you for your participation!** | | |

| **ENDOMETRIOSIS** | | |
| --- | --- | --- |
|  |  | |
| When were you diagnosed with endometriosis? | | |
|  |  | |
|  | | |
|  |  | |
| What type of endometriosis do you have? |  | |
| ☐ Endometrioma | ☐ Superficial endometriosis | |
| ☐ Deep endometriosis | ☐ Others : | |
|  |  | |
|  | | |
|  |  | |
| Do you have adenomyosis? | ☐ Yes | ☐ No |
|  |  | |
|  |  | |
| **The questionnaire is now over.**  **Thank you for your participation!** | | |
